# Molecular Modeling Investigations of Sorption and Diffusion of Small Molecules in Glassy Polymers

**DOI:** 10.3390/membranes9080098

**Published:** 2019-08-08

**Authors:** Niki Vergadou, Doros N. Theodorou

**Affiliations:** 1 Molecular Thermodynamics and Modelling of Materials Laboratory, Institute of Nanoscience and Nanotechnology, National Center for Scientific Research Demokritos, Aghia Paraskevi Attikis, GR-15310 Athens, Greece; 2 School of Chemical Engineering, National Technical University of Athens, GR 15780 Athens, Greece

**Keywords:** polymers, diffusion, transition state theory, sorption, permeability, penetrant, separations, kinetic Monte Carlo, coarse-graining, multiscale modeling

## Abstract

With a wide range of applications, from energy and environmental engineering, such as in gas separations and water purification, to biomedical engineering and packaging, glassy polymeric materials remain in the core of novel membrane and state-of the art barrier technologies. This review focuses on molecular simulation methodologies implemented for the study of sorption and diffusion of small molecules in dense glassy polymeric systems. Basic concepts are introduced and systematic methods for the generation of realistic polymer configurations are briefly presented. Challenges related to the long length and time scale phenomena that govern the permeation process in the glassy polymer matrix are described and molecular simulation approaches developed to address the multiscale problem at hand are discussed.

## 1. Introduction

Molecular modeling and simulation play an increasingly important role in the design of products and processes for addressing the grand challenges faced by contemporary society in domains such as health, energy, food, clean water, and the protection of the environment. This is attested by the dramatic increase in the fraction of research papers in science and engineering that use molecular modeling [1]; by the enhanced role of molecular modeling activities in industry [2]; and by economic analyses of the impact of molecular modeling [3]. Very visible and well-funded national initiatives in the area of materials include modeling and simulation as one of their top priorities (see, for example, Materials Genome Initiative in the United States [4]) or are entirely focused on molecular and multiscale modeling (see, for example, OCTA Project in Japan [5] and European Materials Modeling Council in the European Union [6]).

Molecular simulations of sorption and diffusion in membrane materials have been performed since the late 1980s. This is understandable, given that membrane separations constitute a very dynamic and rapidly growing field of modern technology. Early molecular dynamics simulations were instrumental in elucidating atomic-level mechanisms, e.g., of elementary jumps of gaseous penetrants executed between clusters of accessible volume in a glassy polymer. In the 1990s the basic methodology for predicting sorption isotherms and diffusion coefficients from atomistic simulations was established and validated on well-defined model systems. This methodology is exposed in several past reviews of permeation phenomena in amorphous polymers [7,8,9]. Contemporary simulation efforts have expanded to address permeation phenomena in more sophisticated materials of complex chemical constitution, nanostructured and hybrid organic-inorganic systems. As the application of conventional molecular simulation algorithms is often too computationally demanding for such systems, there is increasing interest in multiscale modeling methods based on systematic coarse-graining of the molecular representation. For some categories of new membrane materials whose synthesis has taken off only recently, such as Metal Organic Frameworks, one sees simulation work going hand-in-hand with, and even guiding, experimental efforts. High throughput computational screening for the identification of new nanoporous material structures that best satisfy the requirements of specific gas separation, sequestration, or storage applications has emerged [10]. Machine learning techniques are starting to be used both in the development of more refined quantum mechanics-based classical force fields (or coarse grained ones based on the atomistic representation) for conducting simulations and in mining the copious data generated by molecular simulations for materials design.

**Figure 1:**
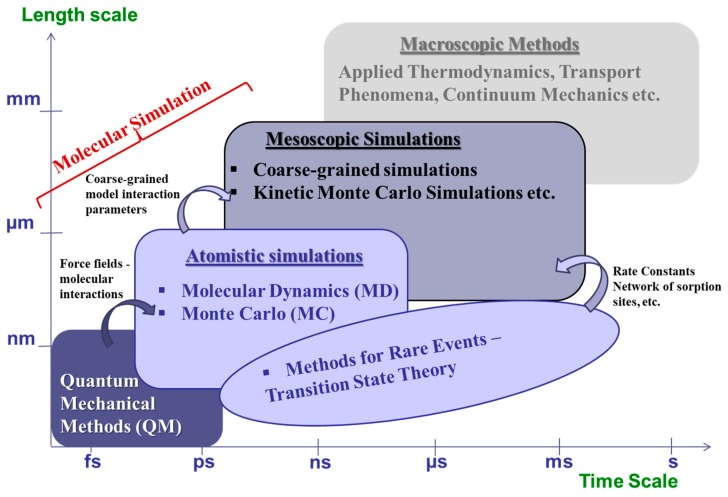
Molecular simulation methods at multiple length- and time scales. Hierarchical multiscale simulations utilize information extracted from the previous scale as input for conducting molecular simulations at longer length and time scales.

In this article we present a brief review of molecular modeling and multiscale simulations (Figure 1) of sorption and diffusion of small molecules in glassy polymers, a broad category of materials which continue being a workhorse of selective membrane separation technology. The treatment is concise and certainly not intended to be all-encompassing. Emphasis is laid on work conducted in the last couple of decades. An extensive reference list is provided, to which the interested reader can resort for details.

The structure of the article is as follows: in Section 2 we present an introduction to basic concepts and discuss equilibrium and non-equilibrium molecular dynamics and Monte Carlo techniques for simulating polymers and sorption and diffusion of small molecules therein. Methodological aspects of the simulations are addressed very briefly—they have been presented extensively in earlier reviews. Emphasis is laid on multiscale methods that incorporate infrequent event analysis of elementary diffusive jumps and on coarse-graining/reverse mapping for the generation of realistic model configurations in which to study permeation phenomena. Both these aspects are necessary for predicting the separation performance of dense glassy polymer matrices, especially if their chemical constitution is complex. Section 3 discusses coarse-grained approaches to the simulation of sorption and diffusion phenomena in polymers per se. The approaches discussed have been designed to address long length and time scale phenomena by cutting down on the computational cost relative to full atomistic analyses; they include methodologies that incorporate mesoscopic kinetic Monte Carlo techniques that utilize information from atomistic simulations (Figure 1) and coarse-grained molecular dynamics simulations accompanied by appropriate time mapping. Some mechanistic aspects of sorption and transport revealed by recent simulation work are presented in Section 4. Emphasis in this Section is laid on the characteristics of the penetrant’s hopping diffusional mechanism, on swelling and plasticization of glassy polymer matrices by compressed CO_2_ and on mixed gas sorption and diffusion phenomena in polymer glasses. Finally, Section 5 briefly pinpoints challenges that still have to be met, especially in conjunction with new polymer-based membrane materials, and reflects on the future outlook of molecular simulations of sorption and diffusion phenomena in dense amorphous polymeric membranes.

## 2. Background and Methodology

During the membrane separation process the penetrant molecules are transferred from an upstream fluid phase to the polymer membrane where they dissolve on the membrane surface (sorption phase) and then diffuse through the polymer matrix, exiting eventually at the downstream fluid phase (desorption). The permeation process through dense polymer membranes is described by the widely known solution-diffusion mechanism, considering a thermodynamic process (sorption) and a kinetic process (mass transport). Mass transport phenomena are governed by random molecular motion and permeation is driven by the presence of chemical potential gradients. In practical formulations the gradient of concentration is used instead of the gradient of chemical potential. In a dense polymeric membrane, permeation of a single component is often described locally by the empirical Fick’s laws [11]:

(1) J=−D∇C

(2) $$\frac{\partial C}{\partial t}=D\nabla^{2}C$$

where C is the concentration of the diffusing component, D is the diffusion coefficient, and t is the time. The concentration profile inside the membrane is considered to reach a steady state after a certain time. Assuming that D is concentration-independent, which is typically the case at low concentrations, Equation (1) can be integrated to [12]:

(3) $$J=D\frac{C_{U}-C_{D}}{l}$$

where l is the membrane thickness and $C_{U}$ and $C_{D}$ are the upstream and downstream permeant concentration, respectively. Equation (3) can be also expressed in terms of fluid partial vapor pressures or fugacities, $p_{U}$ and $p_{D}$ on the two sides of the membrane:

(4) $$J=P\frac{p_{U}-p_{D}}{l}$$

in which P denotes the permeability coefficient. At low concentrations in the Henry regime and considering a constant diffusion coefficient over a range of pressures, the permeability of a membrane to a specific kind of penetrant molecules can be expressed as the product of solubility coefficient, S, and diffusivity, D, of the penetrant in the membrane:

(5) P=DS

where the solubility is considered as the ratio of concentration at a specific pressure to the pressure.

The ideal permselectivity of a membrane material for two penetrant components (*i* and *j*) is defined as the ratio of permeabilities for each pair of species in pure:

(6) $$a_{ij}=\frac{P_{i}}{P_{j}}$$

The ideal permselectivity can be estimated as the product of diffusivity selectivity and solubility selectivity under the assumption that each penetrant component is not affected by the other:

(7) $$a_{ij}=\frac{D_{i}}{D_{j}}\;\frac{S_{i}}{S_{j}}$$

**Figure 2:**
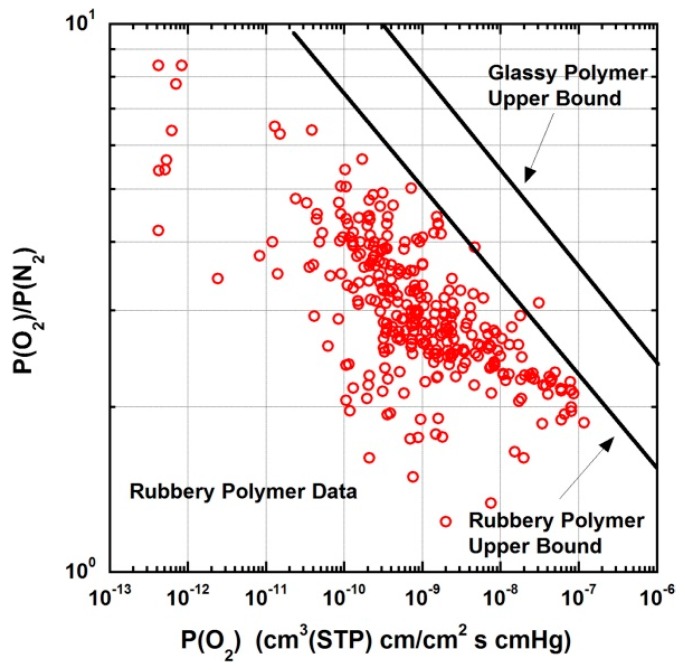
Permselectivity performance of glassy polymers in comparison to rubbery polymers for O_2_/N_2_ separation. Adapted with permission from [15].

A high performance separation membrane [13] is expected to exhibit high selectivity characteristics combined, simultaneously, with satisfactory, or even, ideally, high permeability to one of the penetrant components. The selectivity performance of membrane materials for gas separations is limited by an upper bound. This empirical limit originates from the fact than an increase in permeability of the more permeable component is accompanied by a decrease in the separation ability. If $P_{i}$ is the permeability of the more permeable gas, the upper bound [13,14] is determined as a line in the log-log plot of the relationship $P_{i}=k{\left(\frac{P_{i}}{P_{j}}\right)}^{n}$, where n is the slope of the line, above which no data exist. Data near or on the upper bound correspond almost exclusively to glassy polymers and this is attributed primarily to the higher solubility coefficients in glasses compared to rubbery polymers due to the increase of free volume below the glass transition temperature (*T*_g_), and in some cases partially to a better diffusion selectivity of glassy polymers in comparison to the rubbery ones. In Figure 2, data on selectivity for O_2_/N_2_ separation are plotted as a function of O_2_ permeability for rubbery polymers and the upper bounds for both rubbery and glassy polymers [15,16] are shown that exhibit about an order of magnitude difference in favour of the separation performance of glassy polymers.

Molecular simulation methods have been developed and are employed to unravel the microscopic mechanisms that are responsible for sorption and transport phenomena in glassy polymeric membranes and to predict their macroscopic permeability and selectivity properties. A compact summary of some representative molecular simulation methods and their applicability is provided in Table 1.

### 2.1. Molecular Dynamics

Molecular dynamics (MD) [17,18,19,21] is based on the solution of the classical equations of motion of a system in a microscopic representation via numerical integration. During an equilibrium MD simulation the evolution of the multiparticle model representing the system is followed over time, and thermodynamic and dynamical properties are computed as averages over its trajectory. MD is widely used for equilibration and property prediction in computational materials science using atomistic or coarse-grained, fully flexible or constrained model systems and a number of statistical ensembles. MD is demanding in terms of computational time and a lot of effort has been expended in the direction of reducing these demands by implementing for example domain decomposition methods for the parallelization of MD algorithms and by developing multiple time step integration schemes [50,51] that allow the use of longer time steps for the interactions that change at a slower rate. The phase-space points visited along a long MD trajectory constitute a representative sample of the equilibrium ensemble dictated by the macroscopic constraints (e.g., constant number of particles, volume, and energy) under which the trajectory was generated, allowing the estimation of thermodynamic properties as ensemble averages. Despite progress in algorithmic developments, the extremely broad range of time scales that characterize the various modes of motion in macromolecular systems, especially in the glassy state (from tens of femtoseconds for covalent bond vibrations to years for physical aging), renders MD an insufficient method for properly equilibrating and correctly sampling the dynamics of these systems or the transport of small molecules therein.

The non-equilibrium MD (NEMD) methodology has been employed in an effort to accelerate penetrant diffusion. Here external forces are applied that retain the simulated system away from equilibrium and enable the determination of the induced flux that develops in response to the imposed forces. For small external forces, the transport properties under steady-state conditions can be directly extracted from the ratio of the flux to the imposed force, as the system is weakly perturbed, remaining in the linear response regime [52,53]. NEMD has been applied for the study of transport phenomena in amorphous polymers. Müller-Plathe et al. [54] were the first to employ NEMD for the study of the transport properties of gases in polyisobutylene. No substantial computational gain compared to equilibrium MD simulations was detected in their work within the linear response regime. Van der Vegt et al. [55] implemented NEMD for the simulation of penetrants at low concentrations using a harmonic external potential and determining the linear regime by a single simulation. NEMD has also been used for the investigation of the permeation mechanism in microporous polymers under the limitation of very small fluxes observed for small external forces [56]. Recently, Anderson and co-workers [57] employed NEMD to examine gas transport in the interfacial region of three polymeric systems (polyethylene, poly (4-methyl-2-pentyne) and polydimethylsiloxane), incorporating in the prediction of penetrant permeability and diffusivity the effect of the chain oscillation amplitude and of the accessible cavity fraction in the polymer matrices.

### 2.2. Monte Carlo

Monte Carlo (MC) simulation [18,21] of a microscopic model aims at generating a large number of configurations sampling in an asymptotic manner the probability density of a statistical mechanical ensemble under specific macroscopic conditions. During an MC simulation, each state is generated depending only on the one that precedes it, by implementing an attempted elementary move that may be accepted or rejected [20] based on the energetics and according to specific selection criteria; the latter are designed to follow the principle of microscopic reversibility (or detailed balance) in the generation of the Markov chain sequence of configurations. The attempted moves often involve a very small subset of degrees of freedom, implementing translations and rotations, insertions, deletions, or swaps of molecules, or volume fluctuations of the entire model system. MC is a stochastic technique that does not account for the system’s time evolution and therefore cannot be used for the study of dynamics. This stochastic character of MC, though, coupled with appropriately designed moves, enables the equilibration and sampling of long-chain macromolecular systems orders of magnitude more efficiently than with MD, opening the way to addressing long length scale phenomena.

**Figure 3:**
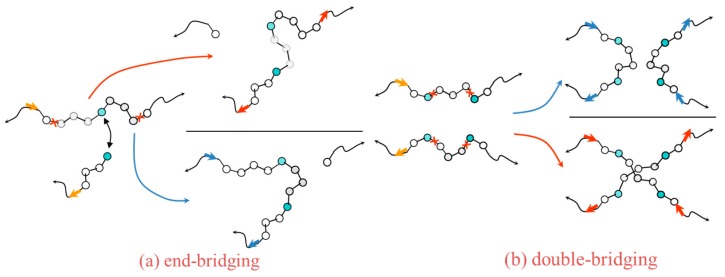
Illustration of the (**a**) end-bridging and (**b**) double-bridging MC moves designed to be implemented for polymeric chains with directionality in their chemical structure. In each case, the moves shown by blue arrows are possible. Intramolecular double-bridging MC moves cannot be performed in the case of directional chains.

For the simulation of polymeric systems sophisticated moves [24,27,58] have been introduced, including configurational bias (CBMC) [59,60,61], concerted rotation (CONROT) [58,62], reptation and various connectivity altering moves such as end-bridging (EBMC) [22,23] and double-bridging (DB) [26]. During an end-bridging move, a chain is divided in two pieces via the excision of a trimer segment located in the chain’s inner part as the chain is “attacked” by the end of another chain that lies in close proximity. One piece of the initial chain is then connected to the attacking end by reconstructing the trimer segment that was originally excised. In similar spirit, a double-bridging move is implemented by excising two trimer segments from two individual neighboring chains that are again reconstructed to bridge the remaining chain parts, forming two new chains of the same length as the original ones, but in a completely different conformation. In Figure 3, end-bridging and double bridging MC moves are illustrated for the case of chains with directionality in their macromolecular architecture, in which only the moves depicted with the blue arrows are allowed to be implemented for the chemical structure to be preserved. Using these moves it is possible to equilibrate at all length scales polymer melts with molar mass distributions comparable to those encountered in membrane applications. Equilibrated melt configurations obtained in this way constitute excellent starting points for generating glassy amorphous polymer configurations through cooling. MC methods are often implemented for the study of sorption in membrane materials (see Section 2.3).

### 2.3. Methods for the Molecular Simulation of Penetrant Sorption

The methods that are used within the context of penetrant sorption mainly involve the implementation of Grand Canonical Monte Carlo (GCMC) and Gibbs Ensemble Monte Carlo (GEMC) [63]. In GCMC the chemical potential, the volume and the temperature are kept constant, while the number of particles changes during the simulation, fluctuating around an equilibrium value. During the GCMC simulation the polymer system is considered to be in contact with a penetrant reservoir at specified temperature and chemical potential, with which it exchanges energy and particles. The trial moves in this case are translations, insertions, and deletions of particles. 

GEMC enables the study of phase equilibria in one simulation by simultaneously incorporating two individual simulation boxes between which there is no interface. The attempted moves in this case involve particle translation and rotation, volume change (usually in a manner such that the total volume is preserved) and random particle swaps between the two simulation boxes implemented to simulate phase equilibrium of the two phases under the same temperature, pressure and chemical potential conditions. In systems containing a nonvolatile condensed (e.g., polymer) component at equilibrium with a pure or mixed gas phase, the solubilities of the gaseous permeants can be calculated using MC in a hybrid isothermal-isobaric and grand canonical ensemble [9] that does not require explicit simulation of the gas phase. This is termed the *N*_1_*f*_2_...*f_n_PT* ensemble and keeps fixed the number of molecules of the condensed component, *N*_1_, the fugacities of the gas components, *f*_2_,...,*f_n_*, as well as the system temperature and the pressure. Note that only *n* of the quantities *f*_2_,...,*f_n_*,*P*,*T* are independent.

In some cases the acceptance probability of the attempted insertions and deletions in the above MC schemes is very low, such as in the case of low-accessible volume polymer matrices, very strong penetrant/polymer interactions, or bulky penetrant molecules. Semigrand canonical ensemble MC [22,64,65] can be applied in this type of binary or multicomponent mixtures, during which identity exchange trial moves among the various species is attempted. Considering, for example, a binary system consisting of species A and B, MC moves are attempted that involve conversion of one particle of species A to species B or vice versa. The change in free energy (difference of chemical potentials) brought about by such a change in the chemical identity is taken into account in the acceptance of the move.

At low pressures, the Henry’s law constants can be calculated using the Widom insertion test particle method [66]. The method involves insertion of a ghost penetrant molecule in the polymer matrix at random positions and orientations and calculation of the interaction energy of the inserted molecule with the matrix. The latter is used subsequently for the determination of the excess chemical potential $\mu^{\mathrm{ex}}$ of the test molecule. The solubility of the molecule in the polymer matrix can be calculated as:

(8) $$S=\frac{22,400{\;\mathrm{cm}}^{3}\left(\mathrm{STP}\right)}{\mathrm{mol}}\frac{1}{RT}\exp\left(-\frac{\mu^{\mathrm{ex}}}{RT}\right)$$

with STP corresponding to a temperature of 273.15 K and a pressure of 101.325 kPa and the solubility obtained in units of ${\mathrm{cm}}^{3}\left(\mathrm{STP}\right)/\left({\mathrm{cm}}^{3}\mathrm{polymer}\;\mathrm{Pa}\right)$. A large number of test particle insertions are implemented in well equilibrated configurations of the polymer systems obtained from MD or MC simulations. The method has been widely applied for the estimation of low concentration sorption of small molecules in polymer systems [7,43,44,45].

Sorption isotherms can also be extracted utilizing iterative schemes that involve MD simulations of the polymer-penetrant system at constant composition, calculation of the excess chemical potential and gas fugacity and carrying out new MD simulations at a corrected pressure until convergence is reached [46,47].

More elaborate computational techniques are often necessary in cases that involve sorption of large penetrants, high pressures or strong penetrant-polymer interactions, especially at low temperatures. These methods include configurational bias insertions [67], which are used for the bond-by-bond insertion of multi-atom solutes for which Widom test particle insertion is not successful; thermodynamic integration [48,49] during which a small solute is included in a dense system gradually using progressively increasing coupling parameters λ for the solute-matrix interactions and conducting a series of simulations; expanded ensemble techniques [68,69] that also involve a stepwise insertion or deletion of the solute and the implementation of free energy perturbation within a single simulation; excluded-volume map sampling [70,71,72,73] and grid search methods [74,75] for efficient sampling in dense systems by blocking regions in which solute insertions are not feasible; the test particle deletion method [76,77] which constitutes an inverse of the Widom method for the calculation of the chemical potential of particles and multisite solutes; extended ensemble MD [78] during which a dynamic coupling parameter is used for the solute-polymer interactions that enhances efficient sampling of the system’s phase space; the scission-fusion MC method [67] for the determination of solubility of oligomer solutes in polydisperse polymeric systems of the same chemical constitution as the solute; grand canonical MD [79] that is implemented for vapor-liquid equilibria and sorption in rigid polymer matrices by conducting two individual simulations in different ensembles without the requirement of particle exchanges between the phases; and fast-growth thermodynamic integration [80] where the chemical potential is calculated efficiently from a number of independent thermodynamic integrations for the estimation of sorption of large molecules in dense matrices.

### 2.4. Molecular Simulation Methods for the Study of Infrequent Events

The wide range of time scales that are involved in the relaxation of various modes of motion in polymeric systems renders their simulation a very complicated task. At temperatures near or below the glass transition temperature (*T*_g_), in particular, the polymer matrix becomes almost static, prohibiting the redistribution of accessible volume. This has profound effects on the diffusion of a small penetrant molecule in a selective glassy polymer. The penetrant spends most of its time executing fast local motions while trapped within pockets of accessible volume, or “sorption sites,” within the polymer matrix. Penetrant jumps between sorption sites are infrequent events [8] and cannot be properly sampled by performing even extensively long MD simulations. Transitions from one cavity to another occur rarely and randomly, and generally involve not only translational motion of the penetrant, but also local rearrangements in the polymer matrix that instantaneously open up passages between neighboring pockets of accessible volume.

Transition state theory of infrequent events (TST) [81,82,83] provides appropriate means for the study of systems that are characterized by time scale separation phenomena and crossing of energy barriers that are much higher than the thermal energy, $k_{B}T$. The dynamical evolution of such systems consists of long periods of vibrational motion within a potential energy basin; transitions between different basins (states) are infrequent, corresponding to average times of many vibrational periods. First order rate constants [84] enable the quantification of the average waiting times (which may be very broadly distributed) that elapse between specific transitions occurring and can be calculated invoking TST. The probabilities of occupancy of two states A and B that are separated by a high energy barrier evolve according to a master Equation:

(9) $$\frac{dp_{A}}{dt}=-k_{A\to B}p_{Α}+k_{Β\to Α}p_{Β}$$

(10) $$\frac{dp_{Β}}{dt}=-k_{Β\to Α}p_{Β}+k_{Α\to Β}p_{Α}$$

where $p_{Α}$ and $p_{Β}$ are the probabilities of occupancy of states A and B, respectively, and $k_{A\to B}$ the rate constant for a transition from state A to state B.

**Figure 4:**
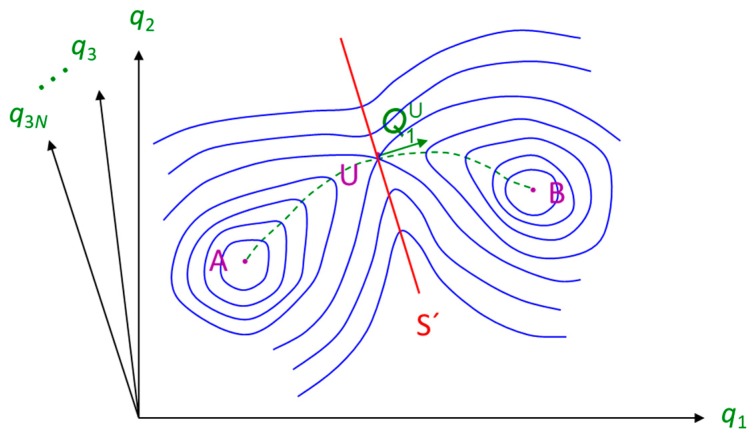
Trajectory, in 3*N* dimensional coordinate space **q**, that passes from one basin to another, crossing a (3*N*-1) dimensional “dividing surface” separating the two basins. Solid lines correspond to a (3*N*-1)-dimensional hypersurface of constant energy V, $Q_{1}^{U}$ is the coordinate along the negative curvature of V(q) (reaction coordinate) and $S^{\prime }$ is a (3*N*-1)-dimensional hypersurface normal to the reaction coordinate, constituting the dividing surface between the states A and B.

Implementing TST for the study of a penetrant jump in a polymer matrix involves the determination of transition states between sorption states (Figure 4) as well as of the pathways followed by the system while passing from one state to another [8,9]. In a glassy polymer matrix there is a large number of regions in which a penetrant molecule may reside and there is at least one transition state between any two states. TST enables the calculation of rate constants along individual pathways based on the probability of the system to be on the dividing surface between two states compared to the probability of its initial state, neglecting the existence of any re-crossing events. From a mathematical point of view, a transition state is a first-order saddle point of the potential energy in the polymer-penetrant multidimensional configuration space, i.e., a point where the potential energy gradient equals zero and there is one negative eigenvalue of the Hessian matrix of second derivatives. The jump pathway can be considered as the lowest energy path (around which actual crossing trajectories would fluctuate) that connects two neighboring local minima passing through the transition state and is usually determined by calculating the Intrinsic Reaction Coordinate (IRC) [85]. The general TST expression for the calculation of the associated rate constant is given by:

(11) $$k_{A\to B}^{TST}=\frac{k_{B}T}{h}e^{\left(-\frac{G^{‡}-G_{A}}{k_{B}T}\right)}$$

with $G^{‡}$ and $G_{A}$ denoting the Gibbs energy of the system lying on the dividing surface and in the initial state, respectively. Using a harmonic approximation for the potential energy and considering the quantum mechanical vibrational partition function, the rate constant can be written as [32]:

(12) $$k_{A\to B}^{TST}=\frac{k_{B}T}{h}\frac{\prod_{a=1}^{f}\left[1-\exp\left(-\frac{h\nu_{a}}{k_{B}T}\right)\right]}{\prod_{a=2}^{f}\left[1-\exp\left(-\frac{h\nu_{a}^{‡}}{k_{B}T}\right)\right]}\exp\left(-\frac{V^{‡}-V_{A}}{k_{B}T}\right)$$

where $\nu_{a}$, $V_{A}$ and $\nu_{a}^{‡}$ $V^{‡}$ are the vibrational frequencies and potential energy at the initial state and at the transition state, respectively. If re-crossing events are important in the process, then a dynamical correction factor to $k_{A\to B}^{TST}$ has to be calculated and taken into account [82,86,87].

Gusev et al. [29] were the first to systematically apply TST for the study of diffusivity of spherical probes in rigid minimum energy glassy polymeric matrices generated using molecular mechanics. Identifying the invocation of a static polymer matrix as a crucial drawback of their study, Gusev and Suter [30] implemented the TST method allowing independent harmonic vibrations of the polymer atoms around their equilibrium positions. The elastic motion of the polymer matrix incorporated in their work involved the harmonic motion of polymer atoms at very short times, thus not taking into account any structural relaxation. The amplitude of the elastic motion was determined by an adjustable parameter $˂\Delta^{2}˃$ that directly affected the extracted rate constants. Within the Gusev-Suter approach, TST calculations were conducted in the three-dimensional space of the spherical penetrant. Their method was computationally efficient and in cases that involved very small penetrants able to extract satisfactory results [7,30,88,89,90].

**Figure 5:**
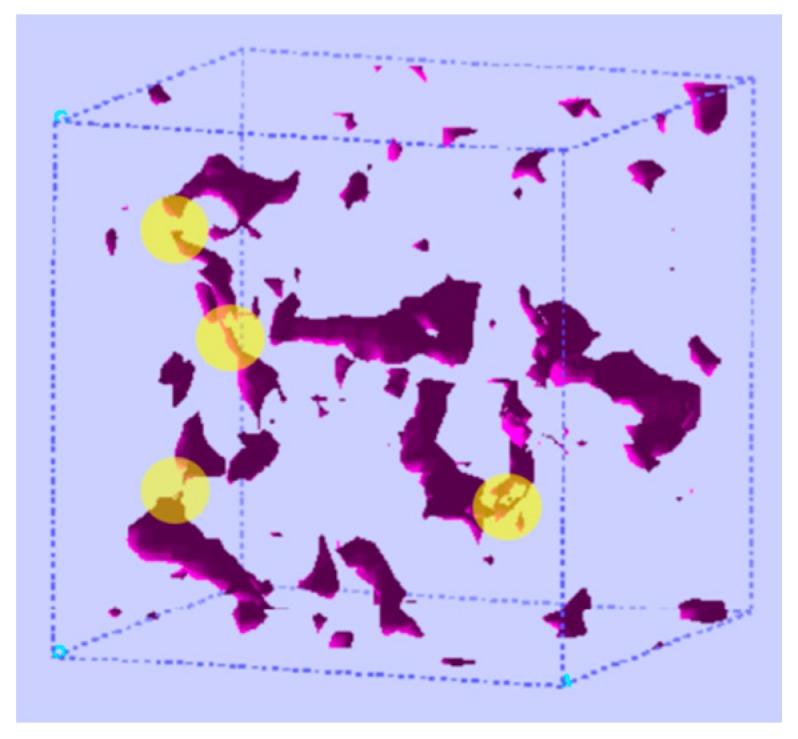
Indicative narrow-necking regions in the accessible volume obtained from geometric analysis, which serve as initial estimates for the transition state search calculations [33]. The simulated polymer is a poly(amide imide) in a cubic box of edge length 28.09 Å with periodic boundary conditions. Atoms are not shown. The dark regions are clusters of accessible volume, as determined with a spherical probe of radius 1.1 Å. Necking regions are identified by probing the same configuration with a smaller probe radius. Some of the necking regions are indicatively highlighted in yellow.

Greenfield and Theodorou [31,32] developed a multidimensional TST methodology in generalized coordinates, in which polymer degrees of freedom are taken into account for the calculation of transition states and diffusion pathways considering a spherical penetrant. The polymer atoms are in this case allowed to move, enabling the realization of local-chain motions that take place during a penetrant passage from one cavity to another. The inclusion of polymer degrees of freedom in the pathway determination becomes very important as the size of the penetrant increases compared to the typical size of clusters of accessible volume in the glassy polymer matrix. Greenfield used a geometric analysis approach [91] for the determination of the accessible volume and its distribution in the polymer matrix to obtain an initial estimate of sorption sites. Narrow-necking regions connecting regions of accessible volume for small spherical probes serve as the starting points for the calculation of the transition states (Figure 5). The reaction path from one state to the other was calculated using a subset of degrees of freedom that were relevant to the specific transition along the pathway. The method was further extended [33,34,92] and applied for the study of polymers with complex chemical constitution in Cartesian coordinates and in the polymer-penetrant multidimensional space using a fully flexible representation for a CO_2_ penetrant.

Rare events can be also studied by implementing transition path sampling methods (TPS) [93,94]. The TPS methodology is used for the calculation of rate constants by sampling dynamical reactive trajectories. Trial trajectories are generated based on a pre-determined transition path, a point of which is randomly selected and the momenta corresponding to it are perturbed. The acceptance probability of the generated trial trajectories can be tuned by the amplitude of the momenta perturbation, with the acceptance increasing for small perturbations.

A number of TPS schemes have been developed to address acceptance probability issues [93,95,96,97,98]. TPS generally enables the determination of realistic pathways at finite temperatures, but is computationally intensive. It has been implemented for the study of a wide range of infrequent events, such as nucleation [99,100,101,102], protein folding and conformational changes [103,104,105,106], glassy dynamics [107], reactions [108,109,110] and penetrant diffusion in non-amorphous materials [111,112,113]. The use of TPS methods for the study of penetrant diffusion in polymeric systems is scarce and involves the study of diffusion of water in a glassy hydrophilic polymer [35]. TPS implementation in this case directly depends on the limited initial transition pathways extracted by MD simulations for the systems under study.

### 2.5. Interactions and Generation of Realistic Structures

Accurate predictions of permeability properties in glassy polymeric systems largely rely on the generation of configurations that are able to resemble the real polymer matrix as well as on the reliable representation of the molecular system under study and the mathematical description of its interactions. A particle-based model may be fully atomistic (AA—all atom); united atom (UA), in which hydrogen atoms are considered to form a single interaction site together with the heavy atom to which they are connected; or coarse-grained (CG), consisting of interacting moieties that are derived by lumping together several atoms. The potential energy function in atomistic models is often derived empirically by fitting the structure, volumetric, thermal, and phase equilibrium properties of small molecule analogues, but also from ab initio electronic structure calculations. It usually consists of a bonded and a nonbonded term, that correspond, respectively, to interactions between atoms that are chemically bonded and to interactions between atoms that are not connected with chemical bonds and may belong to the same or to different molecules. Bonded interactions include contributions to the energy from deviations of chemical bond lengths and bond angles from their equilibrium values and also contributions related to dihedral angles and improper torsions. The non-bonded part includes an electrostatic term, related to the interactions between partial atomic charges (and in general between permanent multipoles), and a van der Waals term. The latter comprises pairwise interactions based on dispersion (attractive) forces due to correlations between electron clouds in different atoms; repulsive (excluded volume) interactions resulting from the overlap of electron clouds of individual atoms at very small distances; and, in some instances, polarization contributions that are also attractive and stem from an induced change in the charges of a moiety owing to the field that it experiences from its neighborhood. The force field parameters for bonded interactions, partial charges, and polarization contributions are often determined by quantum mechanical (QM) calculations, while parameters for the dispersion and excluded volume contributions are typically based on empirical methods or experimental data.

In the general case, the local segment packing that is involved in the penetrant sorption and transport mechanisms, is interconnected with the longer length scale characteristics of the polymeric system [114]. Creation of in silico realistic structures of dense amorphous polymers is a hard and complicated task [34,115]. Amorphous glasses, in particular, are far from equilibrium and are trapped in local energy minima that directly depend on their formation history. Incorporating this fact in molecular simulation strategies for the generation of well-defined and realistic configurations is a great scientific challenge that has still not been adequately addressed.

Several methodologies have been attempted for the generation and equilibration of initial structures of polymers in the melt state [28,114,116,117,118,119,120,121]. Atomistic initial configurations of the macromolecular systems can be rigorously obtained via building the chain in the simulation box bond-by-bond using Flory’s rotational isomeric state model [122], while avoiding excluded volume overlaps based on a modified continuous model for local interactions. These initial configurations are then subjected to energy minimization (molecular mechanics) that can be applied in a stagewise manner [123], removing any remaining atomic overlaps. Molecular mechanics is generally fast but not able to result in an efficient statistical mechanical sampling; nevertheless, it provides a convenient initial configuration for the subsequent implementation of systematic simulation methods towards the generation of realistic glassy polymeric structures. The atomistic structures from molecular mechanics can for example be subjected to equilibration in the melt state using the powerful connectivity altering MC moves [22,23,24,25,26,27,28] or MD or hybrid MC-MD methods. Τhe extracted equilibrated configurations in the melt state have to be subsequently cooled below *T*_g_ [124]. The efficiency of the above scheme is very good for polymer systems of rather simple macromolecular architectures and cannot be easily utilized for polymeric matrices with rigid backbones, strong electrostatic and polar interactions, or bulky side groups.

In the case of polymers with complex chemical constitution, systematic hierarchical approaches are required for the generation of realistic structures which are the starting point for studying the permeability of probe molecules in their bulk. These methods may involve the mapping from the atomistic to a coarse-grained level, equilibration at the coarse-grained level and reverse mapping back to the atomistic representation. The atomistic detail is generally necessary for the study of small-molecule sorption and diffusion phenomena in polymeric matrices. Coarse-graining strategies [115,125,126,127,128,129,130,131] involve the substitution of groups of atoms by single interaction sites, thereby reducing the number of system degrees of freedom, while at the same time maintaining the ones that are important for the description of the mechanisms/processes under study. The CG model invoked in each case is thus intimately related to the macromolecular architecture and the scientific problem at hand. Therefore, the mapping to the CG representation is largely empirical, as there is no unique way to coarse-grain. In order to perform simulations at the CG level, an effective potential for the CG model has to be developed based on the corresponding CG degrees of freedom, that is able to reproduce the key characteristics that need to be retained in the CG simulations. The majority of the CG force fields are developed and parameterized by fitting either in a top-down manner, targeting the reproduction of macroscopic properties [132,133,134,135], or aiming at the reproduction of microscopic properties of the molecular system based on atomistic simulations and experimental findings, following a bottom up approach [136] or using a combination of both strategies [137,138]. Fitting schemes for the parameterization of CG effective potentials include inverse Boltzmann and iterative Boltzmann inversion (IBI) [139], force matching [140,141], inverse Monte Carlo [142] and relative entropy [143,144] methods. In addition to those, interaction potentials between CG moieties can be also determined directly from atomistic interactions of groups of atoms that are mapped onto the CG moieties [145,146], which may also be anisotropic [129]. Hybrid particle-field methods have been also proposed and implemented for the generation of polymer atomic structures [147].

**Figure 6:**
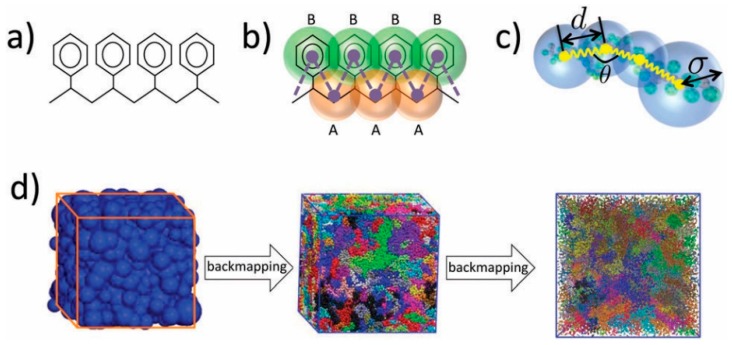
Depiction of a hierarchical backmapping corresponding to (**a**) a united atom representation, (**b**) a moderate CG model consisting of beads of types A and B, and (**c**) a blob-based representation of soft spheres by lumping together a number of beads of representation (**b**). The backmapping procedure is described in (**d**) for the three resolutions. Reproduced with permission from [161].

An ideal CG effective potential would enable the reproduction of target properties and would be also transferable to other thermodynamic state points [137,138,148,149,150,151]. The CG model should also allow, in a straightforward manner, reverse mapping [152,153,154,155,156,157,158,159,160,161] back to representative atomistic configurations (usually on the basis of geometric criteria) of the well equilibrated structures at the CG level. Special measures must be taken for macromolecular structures with complex chemical constitution to avoid potential overlaps or concatenations [162]. The final atomistic configurations from reverse mapping are then subjected to a final relaxation of the system’s local interactions. Figure 6 illustrates a hierarchical backmapping procedure used for the equilibration of polystyrene models at three different representations [161]. The multi-resolution method proposed in this study enables the generation of well-equilibrated high molecular weight polymer configurations that are initially equilibrated at the largest length scale corresponding, in this case, to a soft-blob based representation (Figure 6c). The detailed description of the system is introduced stagewise, first migrating from the soft-blob based to a moderately CG model (Figure 6b) and equilibrating the system locally at this level and subsequently re-inserting the microscopic detail (Figure 6a) and equilibrating again at this stage. The hierarchical backmapping using a sequence of models aims at the efficient generation of realistic long chain polymer melts in a computationally feasible manner, preserving the long-wavelength characteristics of the macromolecular systems under study.

The amorphous polymer configurations to be used for the prediction of solubility and diffusivity properties have to be validated. Validation of amorphous polymer configurations can be realized via direct comparison with experimental measurements over a range of thermodynamic conditions (temperatures and pressures). Such comparison between predicted and measured values can be conducted for a number of properties, e.g., thermodynamic (density, cohesive energy, solubility parameters), structural (pair distribution functions and structure factors from X-ray and neutron scattering), and dynamical (local dynamics of the chain segments and glass transition). Among the structural properties, of particular relevance to permeability is the accessible volume and how it is distributed and connected; predictions can be compared against Positron Annihilation Lifetime Spectroscopy (PALS). It is generally advantageous to generate a large number of configurations, thereby obtaining a sample of representative “trapped” glassy states, and also to verify the stability of the final glassy configurations [9] prior to using them in subsequent simulations.

## 3. Coarse-Graining and Multiscale Approaches in Sorption and Diffusivity Prediction

The broad spectra of length- and time-scales present in macromolecular systems necessitate the implementation of elaborate schemes for the prediction of their properties. Apart from the coarse graining methods related to the generation of realistic initial structures discussed in Section 2.5, hierarchical schemes at multiple length and time-scales have been developed and implemented for the study per se of the permeability of small molecules in polymeric systems.

Transition state theory approaches (see Section 2.4) provide a unique means for calculation of the wide range of interstate rate constants characterizing the elementary jumps executed by a penetrant in an amorphous glassy polymer matrix. By coarse graining to a macrostate level, each penetrant jump can be categorized as an intramacrostate or intermacrostate transition, where a “macrostate” is defined as a collection of basins constructed around neighboring potential energy minima separated by energy barriers that are low compared to the thermal energy $k_{B}T$. At this level of coarse graining, the diffusivity can be calculated considering a Poisson process of successive uncorrelated penetrant jumps between macrostates. For a transition between two macrostates *I* and *J*, the time evolution of the probability that the system is in a macrostate *I* is calculated as follows:

(13) $$\frac{dp_{I}}{dt}=-\underset{J}{\sum }k_{I\to J}p_{I}+\underset{J}{\sum }k_{J\to I}p_{J}$$

where:

(14) $$k_{I\to J}=\underset{i\in I}{\sum }\underset{j\in J}{\sum }k_{i\to j}\frac{p_{i}}{p_{I}}$$

and $p_{I}=\underset{i\in I}{\sum }p_{i}$ and $k_{I\to J}$ is the rate constant for the transition from macrostate *I* to macrostate *J*. A mesoscopic Kinetic Monte Carlo (KMC) simulation [8,163,164,165] is implemented for the calculation of the penetrant diffusion coefficients on the extracted (periodic) networks of macrostates and based on the calculated macrostate-to-macrostate rate constants and sorption probabilities [33,34,92]. This multiscale methodology that combines the TST calculated rate constants and sorption site network with mesoscopic simulations at the macrostate coarse-grained level has been very efficient in the prediction of penetrant diffusivity and in the elucidation of the characteristics of the penetrant’s diffusional motion. Potential artifacts that may arise from the periodic replication of the formed network can be avoided by generating large irregular networks of macrostates using a reverse Monte Carlo (RMC) approach [36]. Analytical approaches for obtaining the diffusivity from penetrant positions representative of each macrostate and from the rate constants between macrostates, taking advantage of the periodic boundary conditions that characterize the model system in each direction, have also been developed [166].

In an effort to extend the time scale limits of the MD simulations, Neyertz and Brown [37,38] implemented a simplified approach that utilizes MD trajectories of mobile gas penetrants in the polymer matrix to extract information that can be used as input in a kinetic Monte Carlo simulation, referred to as trajectory-extending kinetic Monte Carlo (TEKMC). A sequence of configurations visited during a time interval τ is chosen for analysis from MD trajectories of total length of a few nanoseconds. Based on the penetrant positions, the primary simulation box is divided into subcells (of the same size and shape) and the probability matrix of jumps between subcells is extracted. The method is of low computational cost, requiring, however, proper adjustments of the parameters related to the sampling interval τ and subcell grid size [167]. It has been applied for the study of CO_2_ [38] and other small penetrants [37] in fluorinated glassy polymers, leading to predictions in reasonable agreement with experimental measurements. It has also been used for the investigation of gas diffusion in polymer nanocomposites [168] and ion transport in polymer electrolyte melts [169,170,171,172]. The method can only be applied provided that a percolating network of subcells is sampled and that cases of non-linked channels are taken into account in the error of the extracted diffusivity. In order that MD be able to provide an interconnected network of subcells as required by this method, MD simulations are restricted to medium to high penetrant concentrations, in which plasticization effects are usually present [38].

Coarse-grained molecular dynamics (CG-MD) simulations have been also investigated for the modeling of transport of large penetrants in amorphous polymer systems in an attempt to reach the normal diffusion regime. The CG models in this case should be rather detailed, preserving a minimum of important information on the chemical structure of the macromolecular system under study which directly affects the diffusional behavior of the penetrants in the polymer matrix. The CG-MD simulations are accompanied by atomistic simulations for the validation of the CG model used. Kremer and co-workers [39,40] attempted a combined experimental and simulation methodology of this type for the study of the transport properties of ethylbenzene in mixture with polystyrene (PS) using CG MD with different CG representations for the PS. Lin et al. [41] incorporated the MARTINI CG force field [133,173,174] to perform CG-MD of a fragrance agent, octanal, in a polymer film consisting of four components. In their simulations, they studied the concentration and temperature dependence as well as the effect of the presence of water on the effective time scale of the CG simulations and on the diffusional behavior of octanal in the polymeric film. Three CG models have been employed by Kumar and co-workers to study the penetrant diffusion mechanism in flexible and rigid polymers for various penetrant sizes and in a wide range of temperatures [42]. In CG-MD simulations, the simulated behavior does not correspond to the time scales of the dynamics of the real system. “Time mapping” procedures are attempted in this case based on atomistic simulation in order to link the time scales involved in the CG simulations with the time scales of the real dynamics. The effective time scale in the CG-MD is strongly dependent on the mapping implementation, with coarser schemes corresponding to larger effective time scales in the CG simulations, and the calculated mechanisms are directly affected by the loss of chemical detail in the local environment of the penetrant molecule.

## 4. Mechanistic Aspects of Sorption and Transport

The permeation of small penetrants in dense glassy polymeric materials is governed by a number of factors that involve the chemical affinity between the molecule and the polymer as well as characteristics of the glassy polymer matrix. Unlike the mechanism involved in many porous membranes, in which the material participates in the permeation process mostly by the pore structure, in dense amorphous polymeric membranes the polymer is actively engaged in the permeation process.

### 4.1. Sorption

Sorption of solute molecules in a polymer matrix is directly related to both the amount and distribution of free volume in the amorphous phase and to the interactions of the penetrant with the polymer. In the glassy state, the polymeric system is not in thermodynamic equilibrium. Dense polymer glasses are structurally arrested in configurations that depend on the history of their formation (e.g., cooling rate) and have the ability only to fluctuate within a subspace of configurations neighboring the specific local minimum of potential energy in their almost static structure. Structural relaxation in the direction of equilibrium involves the crossing of very high energy barriers that may take place below *T*_g_ in time scales of the order of years, corresponding to physical aging of glassy polymers.

In an equilibrium melt state, the pure polymer as well as the polymer plus penetrant system conforms to a Boltzmann distribution in configuration space. At temperatures near or below the glass transition temperature, the dense amorphous polymer system is no longer in thermodynamic equilibrium. Rather, it is trapped in one among many low energy regions surrounding local minima of the energy, within which it fluctuates thermally. The low energy regions (basins) of the configuration space are separated by high energy barriers that require a lot of time to be overcome. The quenched polymer configurations inherently carry information about the formation history of the glass. Upon cooling, the vitrifying system falls out of equilibrium. The rate of structural relaxation (rate of transitions between different basins in configuration space) decelerates precipitously as the temperature is lowered and becomes much slower than the rate of cooling employed. Redistribution in configuration space cannot keep up with the rate of change in temperature. Thus, the distribution in configuration space becomes arrested, unable to follow the demands of equilibrium, which would dictate passage from higher-energy basins into lower energy ones. The system is trapped within disjoint basins in configuration space, the distribution of these basins being much broader than would be dictated by Boltzmann at the prevailing low temperature. Within each basin however, local reconfiguration is possible; locally, the system can be considered as following a Boltzmann distribution within the confines of the basin. At low penetrant concentrations, the solubility can be studied independently in each disjoint, barrier- surrounded basin and then an average can be taken over individual glassy basins.

**Figure 7:**
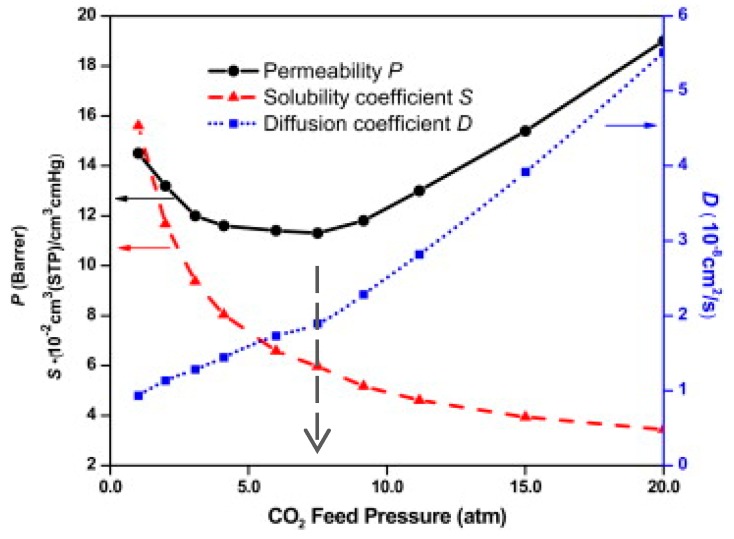
Experimental measurement of CO_2_ permeability, solubility and diffusion coefficient as a function of pressure in a polyimide membrane. The black dashed-line arrow is indicative of the characteristic pressure (“plasticization pressure”) that corresponds to the minimum of permeability. Reproduced with permission from [183].

As a general rule, glassy polymers have a higher sorption capacity than rubbery polymers, with the glassy sorption isotherms exhibiting a concave curvature in contrast to the rubbery ones that are almost linear. During sorption at high concentrations of highly condensable penetrants such as CO_2_, the potential energy barriers that resulted in disjoint subsets of configurations in the dense amorphous glass are altered, enabling a more frequent barrier crossing. This fact leads to volume swelling phenomena that are observed both in glassy and rubbery polymers, due to a penetrant-induced increase in the fractional free volume and to plasticization of the dense amorphous polymer matrix [175,176,177,178,179,180]. These phenomena directly influence the performance of the material as a separation medium [181], enhancing the permeability and diffusivity as the concentration increases. The presence of the penetrant at high concentrations accelerates the segmental dynamics of the chains, resulting in the case of polymer glasses in the reduction of the glass transition temperature due to polymer softening. Plasticization phenomena are described in terms of a characteristic pressure (known as “plasticization pressure”) which is related to a minimum in the gas permeability (Figure 7). Under those circumstances, sorption isotherms of glassy polymers at high pressures may exhibit a rubber type linear behavior, while following a glassy-type curve at lower pressures. Swelling and plasticization behavior of a polymeric system is affected by temperature [182] and this can be considered as an outcome of the interplay of the opposite trends between sorption capacity and chain mobility as a function temperature.

**Figure 8:**
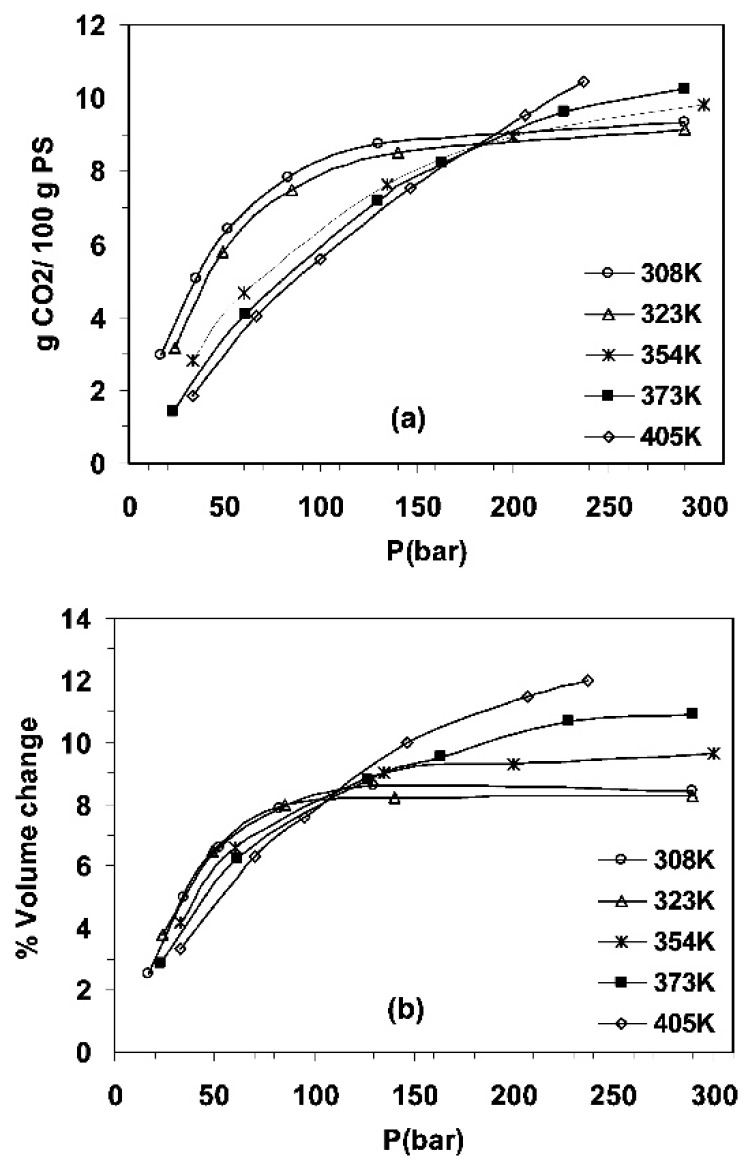
Molecular simulation results on (**a**) isotherms of CO_2_ sorption and (**b**) polymer swelling for polystyrene at various temperatures and pressures. Reproduced with permission from [47].

Sorption and penetrant-induced plasticization and swelling phenomena have been investigated by molecular simulation of bulk polymeric systems [38,183,184,185,186,187,188,189] using either CGMC or implementing iterative multi-stage methods to determine the correct pressure at given penetrant concentration [46,47,190]. Van der Vegt et al. [46,186] have studied sorption of CO_2_ in a polyethylene-like membrane and have identified below *T*_g_ a hole filling mechanism in combination with a finite positive partial molar volume of CO_2_ being responsible for the gas sorption thermodynamics in the glassy polymer. Spyriouni et al. [47] used well equilibrated polystyrene configurations that were obtained implementing a coarse-graining—reverse-mapping scheme [191] that were loaded up with CO_2_ in a wide pressure range. They observed a change in the shape of both sorption isotherms and swelling curves with increasing temperature (Figure 8). In this work, a mapping of the accessible volume was conducted to facilitate efficient loading of CO_2_. The polymer-solute matrices were subjected to NPT MD simulations during which the solute molecules were swapped between accessible volume cavities to achieve an efficient equilibrium repartitioning. The chemical potential calculations were performed using the Direct Particle Deletion method [47]. They also analyzed the polymer segmental relaxation times in the presence of CO_2_ to obtain estimates of the solvent-induced glass transition pressure at various temperatures below the *T*_g_ of pure polystyrene, obtaining good agreement with available experimental measurements. Sorption and dilation effects have been modeled in fluorinated polyimides (6FDA-ODA,6FDA-DPX,6FDA-DAM) [183,192,193], in poly (ether sulfone) (PES) and polysulfone [185] polymers and in copolyimide polymers [188] up to high pressures. Performance and plasticization resistance of an emerging class of polymeric membrane materials called ‘thermally rearranged polymers’ [194,195,196,197,198,199,200,201,202,203,204] that are generated by thermally modifying aromatic polyimide chains, has also been investigated computationally [205,206,207,208,209,210,211] in recent years. These phenomena have been also simulated for high free volume polymers of intrinsic microporosity (PIM) [56,189,212,213].

As the penetrant concentrations increase, the volume dilation phenomena are often semi-permanent and, after desorption of the sorbed gas, part of the additional volume that has been created due to swelling during sorption is preserved, a phenomenon that is often referred to as “conditioning” [214]. A hysteretic behavior is thus observed during subsequent sorption cycles, the extent of which depends on the conditions of the experiment and on the amorphous glass thermodynamic history. Plasticization and conditioning effects are in many cases dependent on the thickness of the amorphous polymer membrane [215]. Physical aging generally takes place more rapidly in thin films. [215,216,217] than in the corresponding thicker ones or the ones of bulk glassy matrices and a different permeability trend is observed during the timescale of the measurements [216]. Physical aging of a glassy polymer matrix is characterized by a decrease in the free volume, leading to a densification [218,219,220] that affects sorption and permeability properties [221,222] as well as the plasticization behavior of the polymer matrix. 

Molecular simulations have been applied incorporating thin membrane models [57,223,224,225,226,227,228,229,230] and explicit gas feed reservoirs that attempt to approximate experimental conditions using MD or NEMD [57]. Such simulations of interfacial phenomena often result in more intense interfacial effects due to the small thickness of the models and the large surface to volume ratio used in simulations, as modeling of dense polymeric films would require the implementation of a very extended surface area corresponding to system sizes that are currently not computationally feasible [223]. In the course of MD of 6FDA-6FpDA fluorinated polyimide membranes [231] using a CO_2_ gas reservoir on both sides of the film, a rapid adsorption was initially observed at polymer-gas interfaces that was subsequently followed by a slower uptake. During the sorption process, swelling effects were detected and the additional free volume regions were occupied at short time by the sorbed penetrants. The same models were also used for the study of the effect of structural isomerism in the plasticization of meta-linked and para-linked polyimides, obtaining a plasticization resistance in the meta-linked isomer [228]. In an effort to study the reversibility of polymer membrane dilation, MD simulation has been applied in an explicit film model of 6FDA-6FpDA polyimide with surfaces in contact with a gas reservoir [229]. Two cycles of sorption and post-degassing phases have been applied and metastable states of enhanced solubility after degassing have been identified that depend on the former swelling stages of the material. The two sorption phases were found to differ at short times, as additional void spaces remain after the first cycle of post-degassing relaxation.

Sorption of mixtures of various gases in glassy polymers is common in membrane separations. The mechanism of permeation varies with the concentration of the mixed gas and the competitive character of the sorption among individual species. At low concentrations, each penetrant type may sorb without being affected by the presence of the other components. For an increasing amount of sorbed gases, one species may be more soluble in the polymer matrix leading to plasticization of the membrane, thus modifying the sorption environment of the other components [175,232]. A more complex behavior may arise if strong interactions are present between one of the components and the polymeric membrane material, or among the various penetrants themselves, thus altering the permselectivity performance of the membrane. The mixed gas behavior generally hinges on the chemical identity of the gas pair and its composition along with the pressure conditions and the thickness of the membrane [233]. The actual permselectivity, $a_{ij}^{*},$ differs from the ideal one (Equation (6)) [232,234] necessitating the investigation of mixed-gas conditions [235,236] along with the ones of the pure gases for the evaluation of the membrane permselectivity performance.

Mixed-gas simulations [57,223,237] have been recently performed for the study of the actual permeability performance of polymeric membranes. Tanis et al. [237] investigated the permeation properties of pure and mixed gas N_2_/CH_4_ in two 6FDA-based fluorinated polyimides and in their block copolymer. For the pure CH_4_ gas, they extracted a similar methane sorption behavior in the three polyimides, accompanied by a linear-type volume swelling. Small differences were observed in the gas solubility coefficients of each species calculated also for the N_2_/CH_4_ mixture in a 2:1 composition, indicating that the permselectivity performance is primarily governed by the kinetics of the gas mixture. MD simulation has been conducted for O_2_/N_2_ simultaneous sorption and separation in a 6FDA-6FpDA polyimide glassy polymer film in the presence of gas reservoirs on each side of the film [223]. 

**Figure 9:**
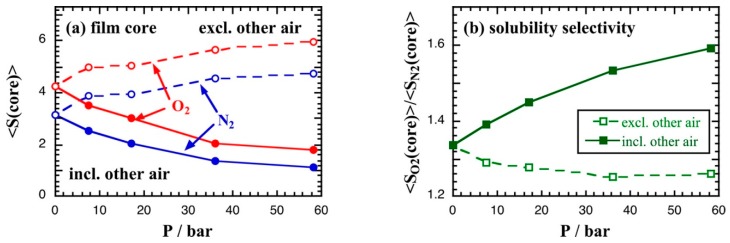
(**a**) Average (dimensionless) solubility as a function of pressure for O_2_ and N_2_ in a 6FDA-6FpDA polyimide glassy polymer film excluding (dashed lines) or including (solid lines) interactions with the other air molecules. (**b**) Solubility selectivity for O_2_/N_2_ separation in the same film. Reprinted with permission from [223].

High initial pressures were applied and very long simulations of 300ns were employed in order for the system to reach equilibrium both in terms of gas concentration and pressure stabilization. The volume swelling effects for the O_2_/N_2_ gas mixture were rather limited and the glassy structure was not significantly affected by the air sorption. Figure 9a depicts the average solubility in the film with increasing pressure both including and excluding the probe interactions with the other air molecules, while Figure 9b shows the solubility selectivity with respect to pressure. Both gases were found to occupy the same low energy voids and the calculated solubility selectivity was extracted in good agreement with single gas permeation experimental measurements [238,239,240,241].

### 4.2. Diffusion

Diffusion through a dense amorphous polymer matrix is a very slow process at temperatures near or below *T*_g_. In dilute systems, the diffusivity of the penetrant depends on the penetrant size and its interaction with the polymer and also on the size and connectivity of the unoccupied volume in the polymer matrix. Below *T*_g_, there is nearly no redistribution of the unoccupied space and the penetrant spends most of its time trapped in one cavity; a jump from one cavity to another rarely takes place when local fluctuations of the polymer segments open a channel that closes again directly after the jump. Diffusion in the dense glassy amorphous matrix consists of a sequence of infrequent events described by a hopping mechanism [242,243] and is directly related to the rate constants that govern the jumps between the neighboring cavities as well as the distance and connectivity of the pre-existing cavities in the polymer matrix.

The long time scales of structural decorrelation in amorphous polymers (even above *T*_g_) result in the existence of an anomalous diffusion regime at short times which is characterized by $˂r^{2}˃\propto t^{n}$, n<1, with $˂r^{2}˃$ being the penetrant’s mean square displacement and t being the time. The penetrant experiences heterogeneities in its local environment and very long time scales are required to enter the Einstein (or Fickian) regime of normal diffusion. Anomalous diffusion can be associated with the wide range of rate constants that govern the penetrant jumps [244,245,246] or with the connectivity of the sorption sites [7,36,43], as well as with the penetrant size [70]. TST-based molecular simulation studies have investigated the diffusion mechanism in glassy polymers, revealing important factors that govern the penetrant’s diffusional behavior such as the range of jump rate constants [32,33], the size, shape and connectivity of the volume clusters that are accessible to the penetrant molecule [33,91], the energetic and entropic contributions to the jump rate constants [9,32,33,247] and the characteristics of the cooperative motion of the polymer chains during the elementary penetrant jumps [32,33,34]. In glassy amorphous solids, a spatially non-uniform dynamics is detected and energy and entropy contributions to the rate constants differ from one region of the polymer matrix to the other. A range of rate constants of the order of 10^−3^–10^3^ μs^−1^ were determined for CH_4_ in glassy atactic polypropylene [32] with a mean jump length of 5 Å. In the TST study of CO_2_ diffusivity in a complex poly (amide imide) system, [-NH-C_6_H_4_-C(CF_3_)_2_-C_6_H_4_-NH-CO- C_6_H_4_(CH_3_)-N(CO)_2_C_6_H_3_-CO-]_n_ [33] the rate constants appear widely distributed, with the majority of transitions being characterized by rate constants of the order of 10^−1^–10^2^ μs^−1^, an average jump length of 5.3 Å and activation energies in the range of 2–11 kcal/mol that fall within range of experimental values of activation energy for diffusion of gases in glassy polymers. The dimensionality of the conducted calculations drastically affects the jump energy barrier, which is significantly decreased by increasing the polymer degrees of freedom in the vicinity of the penetrant included, until an asymptotic behavior in the energy is observed and the energy barrier is no longer influenced by the incorporation of additional polymer degrees of freedom [9,32,33]. Detailed analysis of the CO_2_ diffusion mechanism reveals the existence of a concerted motion of the polymer segments along the transition path that allows the penetrant to pass from one sorption state to an adjacent one. Along the pathway, changes in the orientation of the CO_2_ penetrant take place that are important for the realization of the elementary jumps [33,92].

Increasing the penetrant concentration has a diverse effect on penetrant diffusivity, depending on the specific penetrant-polymer system [232]. The diffusion coefficient remains unaffected if low sorbing penetrants are incorporated in the polymer matrix. If penetrant clustering phenomena are present in the system, then a decrease in diffusivity may be observed. For highly condensable penetrants, high concentrations cause volume swelling effects and increase the free volume and segmental mobility [248], a fact that leads to an acceleration in the mobility of the penetrants in the matrix, resulting in higher diffusivities. Even in this case, though, the jump-type hopping mechanism still characterizes the penetrant diffusional motion [38,227,231] with shorter residence times at these conditions.

Interdiffusion phenomena in the presence of more than one penetrant species have a direct effect on the permeability properties of the polymeric system [249]. In some cases, the presence of the more mobile penetrant has the tendency to accelerate the kinetics of the slower one [235,250]. As a result the diffusional behavior of the faster penetrant is subsequently hindered by an increase in the concentration of the slower one. This behavior was also detected in the computational study of Tanis et al. [237], for fluorinated polyimide homopolymers, according to which actual permeabilities cannot be reliably estimated by the ideal ones. The inverse conclusion was drawn from the simulation of air sorption in a 6FDA-6FpDA polyimide glassy polymer film from which it was determined that mixed gas conditions for O_2_/N_2_ separation can be extracted based on single gas data [223], a fact that is expected for the case of light gases such as O_2_ and N_2_ and in the absence of plasticization and competitive sorption effects [232].

## 5. New Materials, Challenges and Future Outlook

The need to develop new high-performance task specific functional polymer-based membranes necessitates the investigation and use of hybrid and composite materials [251,252,253,254,255,256,257,258,259,260,261,262,263,264,265,266,267,268,269,270,271]. Examples of advanced polymer-based separation media include polymer/inorganic membranes incorporating, for example, zeolites, inorganic particles, or nanoparticles in polymer matrices; carbon nanotube (CNT)/polymer composites; glassy polymerized ionic liquids; polymer/ionic liquid composites; and many other multicomponent combinations of materials in polymeric membranes. Several molecular simulation studies in recent years have focused on the investigation of polymer-based composite and mixed matrix membranes [272,273,274,275]. Ionic liquid/ionic polyimide composites [276,277] have been studied using molecular simulations as gas separation media and the effect of the anion structure on gas permeability and selectivity has been studied and is to be confirmed by experimental measurements. Moreover, polymer electrolyte membranes have been studied computationally for use as clean water and desalination membranes [278] and the transport of ions in polymer electrolytes has been simulated [258] also in the presence of nanoparticles [170,171,279,280]. Gas barrier properties in several mixed matrix organic-inorganic membranes have been studied computationally such as in MOF/polymer composites [281,282,283], in zeolite/polymer mixed matrix materials [284], in polyhedral oligomeric silsesquioxane/polymer systems [230,285] and in polymer/nanotube composites [286].

In terms of molecular simulation, for the reliable prediction of properties and the fundamental understanding of the underlying mechanisms in complex and multicomponent materials, it is crucial that the existing hierarchical methods, described in the previous sections, be extended and generalized so that they can be implemented in a straightforward manner. Additionally, unravelling the interfacial interactions [287,288] and characteristics [289,290,291] in polymer-based composite materials is of great importance for an accurate description of their behavior. Development and implementation of efficient, hybrid and adaptive resolution multiscale molecular simulation methods [125,292,293,294,295,296,297,298] may in many cases be necessary for the systematic study of microscopic behavior at the interfaces in composite materials and mixed matrix membranes and for further advancement of the materials-by-design target. In this direction it would be worthwhile to study many technologically and commercially important polymer-based nanocomposite membranes [299,300,301,302,303,304,305] such as Matrimid-based ones [306,307,308], incorporating a number of potential inorganic fillers. Multiscale molecular simulation strategies in the area of next generation multicomponent nanostructured materials are important in identifying how the fillers can be tailored towards an optimum selectivity and permeability behavior, investigating a number of crucial factors such as the effects of the size and the dispersity of the fillers and nanoparticles in the terminal properties as well as the influence of the morphology and surface treatment on the behavior and stability of the multicomponent membrane. Systematic strategies need to be applied for the study of nanocomposite materials [275,309] and for the modeling of complex membranes as for example dense glassy 3D networks, developing in this case efficient cross-linking algorithms [310]. The challenge of modeling materials with increasing complexity and the high demands in terms of system sizes and in the accuracy of predictions is greatly supported by the constant increase in the computational power in the one hand and on the development of novel efficient new algorithms and methods that need to be implemented to enable the in-depth understanding of the composite materials.

In parallel, artificial intelligence and machine learning approaches [311,312,313,314,315,316,317] appear as very promising in the direction of development of improved atomistic interaction potentials utilizing directly the quantum mechanical scale, or of more accurate CG force fields extracted on the basis of the atomistic detail. In the field of force field development in general, the open challenge of optimized transferable interaction potentials is still to be met.

Apart from design of new materials with controlled properties, molecular modeling can also significantly contribute to the design and optimization of novel separation processes. For example, membrane technologies that are based in pervaporation [318,319,320] are used for the dehydration of water-organic mixtures, the separation of organic compounds from water or for separations in organic-organic mixtures. Molecular simulation using NEMD techniques can be utilized to simulate such a process and aid in the optimization of the applied fluxes.

Membrane technology is of great economic, environmental and industrial importance and is directly connected to many societal challenges that need to be faced world-wide in the years to come. Future sustainability relies on the indisputable need for green technologies and security of the water-food-energy nexus around the globe. Efficient membrane materials design is a prerequisite for the development of advanced green cutting-edge separation and barrier processes and the complementary contribution of novel experimental and computational techniques is pivotal in order for the scientific and engineering community to address the critical environmental and sustainability issues.

## Figures and Tables

**Table 1:** Compact summary of representative computational methods and their applicability for the prediction of solubility and diffusivity in glassy polymers.

Method	Application	Advantages	Disadvantages	Refs.
Molecular Dynamics	Numerical integration the system’s classical equations of motion	Calculation of thermodynamic, dynamic and transport properties	Not able to correctly sample the dynamics of systems that are characterized by a broad range of time scales or rare events	[17,18,19]
Monte Carlo	Stochastic method—generation of a Markov chain sequence of configurations	Efficient in sampling long-chain macromolecular systems when coupled with appropriately designed moves ^‡^	Does not account for the system’s time evolution—cannot be used to study the system’s dynamics	[18,20,21] ^‡^[22,23,24,25,26,27,28]
Transition State Theory	Infrequent events	Determination of rate constants and penetrant jump pathways	Multidimensional TST that accounts for polymer cooperative motion is computationally intensive	[8,9,29,30,31,32,33,34]
Transition Path Sampling	Infrequent events	Determination of realistic pathways at finite temperatures	Dependence on the limited initial transition pathways extracted by MD simulations	[35]
Kinetic Monte Carlo	Mesoscopic simulation of a Poisson process	Calculation of penetrant diffusivity by solving numerically the master equations	Requires information on sorption sites network, rate constants and sorption probabilities determined from the atomistic scale	[8,33,34,36,37,38]
Coarse-grained MD	Simulation of dynamics at a mesoscopic scale	Simulation of longer time- and length- scales	Effective time scales in CG-MD that do not correspond to the real dynamics Loss of important chemical detail that governs the penetrant diffusion mechanism	[39,40,41,42]
Widom Test Particle Insertion Method	Sorption	Low concentration sorption of small molecules	Inefficient for dense systems/large solutes	[7,43,44,45]
Iterative Widom Schemes	Sorption	Determination of sorption isotherms	Inefficient for dense systems/large solutes	[46,47]
Thermodynamic Integration	Sorption	Enhanced efficiency for dense systems	Requires conduction of a series of simulations	[48,49]
